# Therapy of chronic hepatitis C in people who inject drugs: focus on adherence

**DOI:** 10.1186/s12954-021-00519-y

**Published:** 2021-06-30

**Authors:** Sona Frankova, Zuzana Jandova, Gabriela Jinochova, Miluse Kreidlova, Dusan Merta, Jan Sperl

**Affiliations:** 1 Department of Hepatogastroenterology, Institute for Clinical and Experimental Medicine, Videnska 1958/9, 14021 Prague, Czech Republic; 2 Psychiatric Hospital Havlickuv Brod, Havlickuv Brod, Czech Republic; 3 Addiction Centre Prague, Prague, Czech Republic; 4 Institute of Medical Biochemistry and Laboratory Diagnostics, First Faculty of Medicine Charles University and General University Hospital in Prague, Prague, Czech Republic; 5 Cardiothoracic Anaesthesiology and Intensive Care, Department of Anaesthesiology and Intensive Care Medicine, Institute for Clinical and Experimental Medicine, Prague, Czech Republic; 6 First Faculty of Medicine, Charles University, Prague, Czech Republic

**Keywords:** Adherence, Czech Republic, Drug user, Hepatitis C, PWID, Treatment

## Abstract

**Background:**

Intravenous drug use (IVDU) represents the major factor of HCV transmission, but the treatment uptake among people who inject drugs (PWID) remains low owing to a false presumption of low efficacy. The aim of our study was to assess treatment efficacy in PWID and factors determining adherence to therapy.

**Methods:**

A total of 278 consecutive patients starting DAA (direct-acting antivirals) therapy were included, divided into two groups: individuals with a history of IVDU, PWID group (*N* = 101) and the control group (*N* = 177) without a history of IVDU.

**Results:**

Sustained virological response 12 weeks after the end of therapy (SVR12) was achieved by 99/101 (98%) and 172/177 (98%) patients in the PWID and control group, respectively; in PWID group, two patients were lost to follow-up, and in the control group, four patients relapsed and one was lost to follow-up. PWID patients postponed appointments significantly more often, 29 (28.7%) in PWID versus 7 (4%) in the control group, *p* = 0.001. Thirteen of 101 (12.9%) and six of 177 (3.4%) patients in the PWID and in the control group, respectively, missed at least one visit (*p* < 0.01). However, postponing visits led to a lack of medication in only one PWID. In the PWID group, older age (*p* < 0.05; OR 1.07, 95% CI 1.00–1.20) and stable housing (*p* < 0.01; OR 9.70, 95% CI 2.10–56.20) were factors positively contributing to adherence. Contrarily, a stable job was a factor negatively influencing adherence (*p* < 0.05; OR 0.24, 95% CI 0.06–0.81). In the control group, none of the analyzed social and demographic factors had an impact on adherence to therapy.

**Conclusions:**

In PWID, treatment efficacy was excellent and was comparable with SVR of the control group. Stable housing and older age contributed to a better adherence to therapy.

## Introduction

Hepatitis C virus infection (HCV) is a significant source of preventable morbidity and mortality among persons who inject drugs (PWID) [1, 2]. In Western countries, as well as in the Czech Republic, intravenous drug use (IVDU) represents the major factor of HCV transmission, responsible for 50–80% of newly diagnosed cases [3, 4].

The prevalence of HCV infection in the population of PWID is high in comparison with general population and increases with the amount of time of ongoing IVDU [5, 6]. The Czech Republic is a country with low HCV prevalence (estimated prevalence in 2017: 0.5%); however, the rate of anti-HCV-positive PWID is high: 67.5% [4, 7, 8]. This group represents the most important epidemiologic risk of transmission of the virus in the population [1]. Furthermore, treatment of HCV infection not only leads to cure of HCV-positive individuals, which prevents further progression of liver disease, but also represents a critical approach in prevention of onward transmission of the infection [2].

Available epidemiological data show that the numbers of treated individuals among PWID remain low. In the interferon era, only 1–2% of infected PWID were treated and they were excluded from nearly all early clinical trials of HCV treatment [9, 10]. At the present time, 8% are treated in Australia and 7–16% in Canada and the USA [11–13].

Many barriers to HCV care in PWID have been identified; the major obstacle is a lack of treatment facilities suitable and adapted for PWID population [14]. When referred to secondary or tertiary centers, PWID often miss their appointments and they can suffer from stigmatization and face treatment refusal. The refusal or deferral of treatment is based on the false presumption of low treatment efficacy, bad adherence to therapy and a high risk of reinfection. However, several studies supporting evidence of high efficacy in PWID population have recently been published with sustained virological response (SVR, i.e., cure of the infection, defined as negative HCV RNA 12 or 24 weeks after treatment completion) rates exceeding 90% in patients with ongoing IVDU or on opioid substitution therapy [15–17].

In the Czech Republic, PWID are also perceived as a difficult-to-treat population and treatment of HCV in this group is deferred in many HCV-dedicated treatment centers. The aim of our study was to assess HCV treatment efficacy in PWID in comparison with individuals without a history of addiction with a special focus on factors influencing adherence to therapy.

## Methods

All consecutive patients who initiated anti-HCV therapy at the outpatient department of Hepatogastroenterology Department at Institute for Clinical and Experimental Medicine, Prague, Czech Republic, between January 1, 2017, and August 6, 2018, were enrolled in order to evaluate SVR 12 and 24 weeks after the end of therapy. The cohort of patients was divided into two groups:

*PWID group (PWID, N* = *101):* patients with a history of intravenous drug use. Duration of abstinence from drug use did not influence treatment initiation: former, recent and ongoing users complying with the principles of harm reduction were included as well as individuals on opioid substitution therapy.

*Control group (N* = *177):* patients without a history of IVDU, who were treated within the same time period.

The patients' data were extracted from the electronic patient database containing records of all scheduled, postponed and unscheduled patients' visits during the treatment and follow-up period. The recording of medical history at individual visits was based on a uniform template; all the patients were treated only by two physicians of the center (S. F. and J. S.); thus, all the analyzed data were mentioned in the medical records. Adherence to the medical appointments was assessed via the records in the hospital information system electronic diary. Drug accountability was checked at every visit of the center.

### Antiviral treatment choice

All patients in both groups were treated with a combination of direct acting antivirals (DAA); a regimen containing interferon α was administered to none of the patients. All DAA combinations administered to the patients are listed in Table 1. The choice of the treatment regimen and its duration (8 or 12 weeks) was based on the following criteria: reimbursement by Czech Public Health Insurance at the time of treatment initiation, HCV genotype, HCV RNA baseline level, liver fibrosis stage (assessed by vibration controlled transient elastography, Fibroscan^®^, Echosens, Paris, France) and potential drug–drug interactions with concomitant medication.

**Table 1:** Used antiviral treatment

	PWID group (*N* = 101)	Control group (*N* = 177)	*p*
Previous IFN-α treatment	19 (18.8%)	69 (39%)	**0.03**
Direct-acting antivirals combination			
Paritaprevir/ritonavir + ombitasvir + dasabuvir 8 or 12 weeks (75/50/12.5/250 mg, three pills in the morning, one pill in the evening)	41 (40.6%)	83 (48.7%)	**< 0.01**
Sofosbuvir + ledipasvir 8 or 12 weeks (400/90 mg, one pill once daily)	18 (17.8%)	19 (10.7%)	
Grazoprevir + elbasvir 12 weeks (100/50 mg, one pill once daily)	19 (18.8%)	57 (32.2%)	
Sofosbuvir + velpatasvir 12 weeks (400/100 mg, one pill once daily)	18 (17.8%)	10 (5.6%)	
Sofosbuvir + velpatasvir + voxilaprevir 8 or 12 weeks (400/100/100 mg, one pill once daily)	3 (3.0%)	4 (1.4%)	
Other	2 (2.0%)	4 (1.4%)	
Treatment duration			
8 weeks	27 (26.7%)	36 (20.3%)	N. S.
12 weeks	74 (73.3%)	141 (79.7%)	
Use of ribavirin (twice daily according to body weight, 800–1200 mg)	19 (18.8%)	22 (12.4%)	N. S.

*P* value of < 0.05 was considered statistically significant and the results are displayed in bold

*PWID* people who inject drugs group

### Laboratory assessment

HCV RNA was assessed by the Roche COBAS^®^ AmpliPrep/COBAS® TaqMan® HCV Quantitative Test v2.0 (Roche Molecular Systems Inc., Branchburg, NJ, USA) at baseline, at weeks 4, 8 and 12 of therapy and 12 and 24 weeks after the end of therapy. HCV genotype was assessed before treatment initiation using the SIEMENS Versant^®^ HCV Genotype 2.0 Assay (LiPA) (Siemens Healthcare Diagnostics Inc., Tarrytown, NY, USA).

### Compliance with ethical standards

The study was approved by the Ethics Committee of Thomayer's Hospital and the Institute for Clinical and Experimental Medicine, Prague, Czech Republic, and was carried out in compliance with the Helsinki Declaration. The patients’ informed consent was not required by local law because of the retrospective design of the study and the use of data from which the patients’ identification information had been removed.

### Statistical analysis

Treatment efficacy was assessed as intention-to-treat, and all patients were included in the statistical analysis in all timepoints. Continuous variables are presented as means and standard deviations, whereas categorical variables are expressed as frequencies (%). Categorical data were analyzed using the Chi-square test. For continuous data, Student’s *t*-test or the nonparametric Mann–Whitney test were used appropriately. Factors of treatment adherence were examined using multivariate logistic regression analysis. All statistical analyses were two-sided, and *p* value of < 0.05 was considered statistically significant throughout the study. Statistical analysis was performed using the GraphPad Prism version 8.2.1 for Mac, GraphPad Software, San Diego, California, USA, www.graphpad.com and R programming language version 3.2.0 (www.r-project.org).

### Methods to improve adherence to therapy

PWID patients who were referred to therapy by harm reduction services were accompanied by employees of harm reduction centers or by peer workers, especially at the first visit. For all who started antiviral treatment, a 24-h helpline was available for support and consulting their medical condition. The appointments were planned taking into account patient’s lifestyle and acceptability of job absenteeism. We minimized the number of appointments needed for treatment initiation: Blood sampling, transient elastography, ultrasound examination and the appointment with the physician took place within 1 h. The blood draw was possible during the whole of the working day, when needed. Antiviral treatment was initiated immediately when HCV RNA and genotype results were available, ideally at the first visit, usually within 1 week.

All patients obtained advice on medication use with focus on regular drug intake, without skipping doses and the risks of premature termination and treatment interruption. All concomitant medications potentially influencing treatment efficacy were carefully reviewed. The patients were advised how to increase adherence to therapy: reminder alarms in the mobile phone, a link between medication and a regular daily activity (i.e., breakfast, arrival at work). If the patients missed their appointment, they were contacted by telephone or e-mail and the visit was rescheduled for the following day. All visits were planned 3 days before taking the last dose of dispensed medication.

## Results

### Patients’ demographic characteristics

The demographic characteristics of both studied groups are summarized in Table 2. In the PWID group, there were significantly more males and the age of the patients was significantly lower in comparison with the control group. There were a comparable number of foreigners in both groups in whom Czech was not their native language. However, their level of Czech language was satisfactory, they all had a stable job and medical insurance. In the group of PWID, IVDU was taken as the most probable factor of HCV transmission; in the control group, 27.7% of patients were infected via blood transfusion before 1992, and nearly half of the patients in the control group did not report any potential risk factor for HCV infection.

**Table 2:** Demographic characteristics

	PWID group (*N* = 101)	Control group (*N* = 177)	*p*
Male sex	66 (65.3%)	91 (51.4%)	**0.02**
Age (years, median, range)	40 (22–68)	59 (22–87)	**< 0.001**
Foreigners	12 (11.9%)	37 (20.9%)	N. S.
Mode of transmission			
Intravenous drug use	101 (100%)		**< 0.001**
Blood transfusion		49 (27.7%)	
Tattoo		11 (62%)	
Sexual transmission		2 (1.1%)	
Nosocomial acquisition		16 (9.0%)	
Professional exposure		13 (7.4%)	
Men having sex with men		4 (2.3%)	
Unknown		82 (46.3%)	
Abstinence			
Recent or ongoing drug use	19 (18.8%)	N. A	
> 1 year	24 (23.8%)		
> 5 years	58 (57.4%)		
Opioid substitution therapy			
Methadone	2 (2%)	N. A	
Buprenorphine	0 (0%)		
HCV genotype			
1a	34 (33.7%)	13 (7.3%)	**< 0.001**
1b	45 (44.5%)	148 (83.7%)	
3	19 (18.8%)	13 (7.3%)	
Other	3 (3%)	3 (1.7%)	
Baseline HCV RNA (IU/mL, median, range)	763,000 (35–13,500,000)	1,340,000 (912–24,900,000)	**0.04**
Fibrosis stage (Metavir score)			
F0–F1	41 (40.6%)	72 (40.7%)	
F2	27 (26.7%)	29 (16.4%)	**0.02**
F3	13 (12.9%)	14 (7.9%)	
F4	20 (19.8%)	62 (35.0%)	

*P* value of < 0.05 was considered statistically significant and the results are displayed in bold

*PWID* people who inject drugs group

More than one half of the patients in the PWID group (57.4%) declared abstinence from illicit drug use of more than 5 years, and 18.8% of patients self-reported ongoing drug use. Two patients were included in the opioid substitution program with methadone. In the control group, a significantly higher number of patients had previously been unsuccessfully treated with an interferon α-based regimen.

HCV genotype 1b was the most prevalent in both groups, but the genotype distribution differed significantly between groups (a higher frequency of genotypes 1a and 3 in the PWID group). PWID presented with a less advanced liver disease and had lower baseline HCV viral load in comparison with the control group.

### Patients' social characteristics

The data are summarized in Table 3. In the PWID group, a significant number of patients reported imprisonment and more often reported an amateur tattoo. In the PWID group, a significantly higher rate of patients smoked and reported harmful alcohol drinking (a daily dose of more than 20 g and 30 g of alcohol in women and men, respectively) [18], and 6.9% of patients in the PWID group had a history of alcohol addiction therapy. The patients with a dual diagnosis represented 28.7% of the PWID group.

**Table 3:** Social characteristics

	PWID group (*N* = 101)	Control group (*N* = 177)	*p*
Imprisonment	7 (6.9%)	1 (0.6%)	**0.004**
Tattoo	50 (49.5%)	12 (6.8%)	**< 0.001**
Smoking	69 (68.3%)	37 (20.9%)	**< 0.001**
Harmful drinking history	13 (12.9%)	7 (4.0%)	**0.008**
Alcohol addiction treatment history	7 (6.9%)	2 (1.1%)	
Dual diagnosis	29 (28.7%)	9 (5.1%)	**< 0.001**
Reference to treatment			
Infectious diseases specialist	34 (33.7%)	68 (38.4%)	**< 0.001**
Gastroenterologist	25 (24.8%)	67 (37.8%)	
General practitioner/other specialist	11 (10.9%)	30 (17%)	
Addiction specialist, psychiatrist	9 (8.9%)	0 (0%)	
Self-reference	22 (21.8%)	12 (6.8%)	
Job			
Stable	56 (56.3%)	90 (50.8%)	**< 0.001**
Unstable	5 (5.0%)	0 (0%)	
Unemployed	13 (12.9%)	4 (1.4%)	
Retired	1 (1%)	75 (43.2%)	
Disability leave	15 (14.9%)	7 (4.0%)	
Maternity leave	11 (9.9%)	1 (0.6%)	
Education			
Primary	20 (19.8%)	4 (1.4%)	**< 0.001**
Secondary, qualified worker	57 (56.4%)	92 (52.0%)	
Secondary, leaving examination	19 (18.8%)	64 (38.0%)	
University	0 (0%)	15 (8.5%)	
Unknown	5 (5.0%)	2 (1.1%)	
Housing			
Stable	86 (85.1%)	176 (99.4%)	**< 0.001**
Unstable	15 (14.9%)	0 (0%)	
Homeless	0 (0%)	1 (0.6%)	

*P* value of < 0.05 was considered statistically significant and the results are displayed in bold

*PWID* people who inject drugs group

There were 22 PWID patients self-referred to HCV therapy based on information obtained from media or successfully treated friends. The PWID were more often unemployed and had a significantly lower education in comparison with the control group. They more often reported unstable housing, but none of them was homeless.

### Antiviral treatment efficacy

SVR 12 weeks after the end of therapy (SVR 12) which is considered as cure of HCV infection [2] was achieved by 98% of patients in the PWID group. Two non-SVR patients were lost to follow-up. In the control group, the SVR rate was identically 98%, four patients experienced relapse of HCV infection and one patient was lost to follow-up. The SVR 12 rate did not differ significantly between groups (Table 4).

**Table 4:** Treatment efficacy and adherence to therapy

	PWID group	Control group	*p*
SVR 12	99 (98%)	172 (98%)	N. S.
Lost to follow-up	2 (2.0%)	1 (0.6%)	
Relapse	0 (0%)	4 (1.4%)	
SVR 24	89 (88.1%)	163 (92.1%)	N. S.
Lost to follow-up	10 (9.9%)	5 (2.8%)	
Relapse	1 (1%)	9 (5.1%)	
Reinfection	1 (1%)	0 (0%)	
SVR 12 according to adherence			N. S.
100% adherent	69 (100%)	153 (95.6%)	
Non-100% adherent	30 (93.8%)	15 (88.2%)	
Treatment termination on time	99 (98%)	169 (95.5%)	N. S.
Premature treatment termination	0 (0%)	1 (0.6%)	N. S.
Postponed treatment termination	2 (2.0%)	7 (4.0%)	N. S.
Delay (days)			
Patient 1	3		
Patient 2	3		
Patient 3		2	
Patient 4		1	
Patient 5		1	
Patient 6		1	
Patient 7		7	
Patient 8		2	
Patient 9		2	
Missed doses (no. of patients)			
1 dose	0	3	N. S.
2 doses	0	3	
3 doses	2	0	
7 doses	0	1	
Postponed visits (no. of patients)	29 (28.7%)	7 (4.0%)	**< 0.001**
1 visit	17	7	
2 visits	8	0	
3 visits	4	0	
Missed visits (no. of patients)	13 (12.9%)	6 (3.4%)	**0.006**
1 visit	11	6	
2 visits	2	0	
Lack of medication (no. of patients)	1 (1%)	2 (1.1%)	N. S.
Patient 1 (missed doses)	3		
Patient 2 (missed doses)		2	
Patient 3 (missed doses)		2	
Missed visit 12 weeks post-treatment (SVR 12)	2 (2%)	1 (0.6%)	N. S.
Missed visit 24 weeks post-treatment (SVR 24)	11 (10.9%)	2 (1.1%)	**0.004**

*P* value of < 0.05 was considered statistically significant and the results are displayed in bold

*PWID* people who inject drugs group, *SVR* sustained virological response

SVR 24 was achieved by 88.1% of patients in the PWID group, one patient relapsed, one patient got re-infected, and ten patients were lost to follow-up at 24 weeks post-treatment. In the control group, SVR 24 rate was 92.1%, altogether, nine patients relapsed and five were lost to follow-up. The SVR 24 rate did not differ significantly between groups despite the higher number of lost to follow-up patients.

### Adherence to therapy

The data are summarized in Table 4. All but one patient terminated the whole course of 8- or 12-week therapy on time or a few days later. Two and four percent of patients terminated late in the PWID and control group, respectively. In the PWID group, the maximal delay was 3 days and a 7-day delay was reported in one patient in the control group, who interrupted therapy due to diarrhea in ulcerative colitis. Both patients in the PWID group missed three doses of antiviral medication.

PWID patients had a significantly higher number of postponed medical appointments (28.7% patients vs. 4% in the control group, *p* = 0.001); however, postponing visits led in only one PWID patient to a lack of medication and skipped dosing. In the control group, two patients postponed their appointments and ran out of medication. The difference in missed visits in the follow-up period was not statistically significant between groups at week 12 after the end of therapy, whereas PWID patients missed the SVR 24 visit statistically more often (10.9% vs. 1.1%, *p* = 0.004). The achieved SVR 12 according to achieved adherence to therapy differed neither in the PWID nor in the control group.

**Fig. 1:**
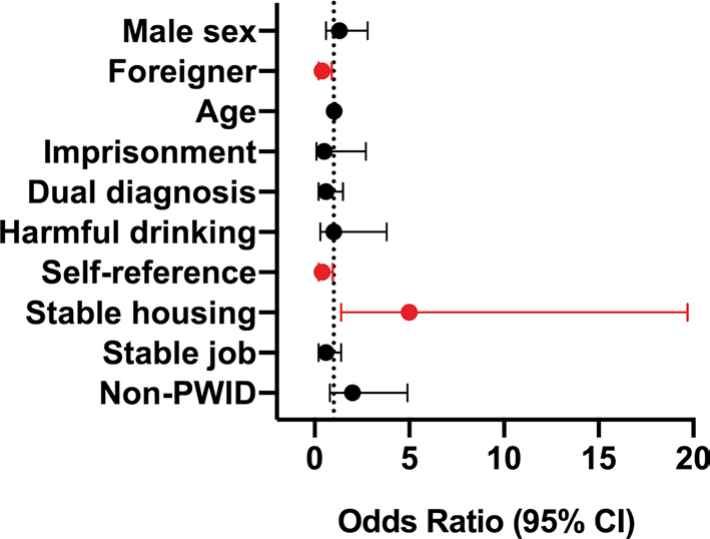
Factors determining adherence to therapy (the whole cohort of patients). *CI* confidence interval, *PWID* people who inject drugs group

**Fig. 2:**
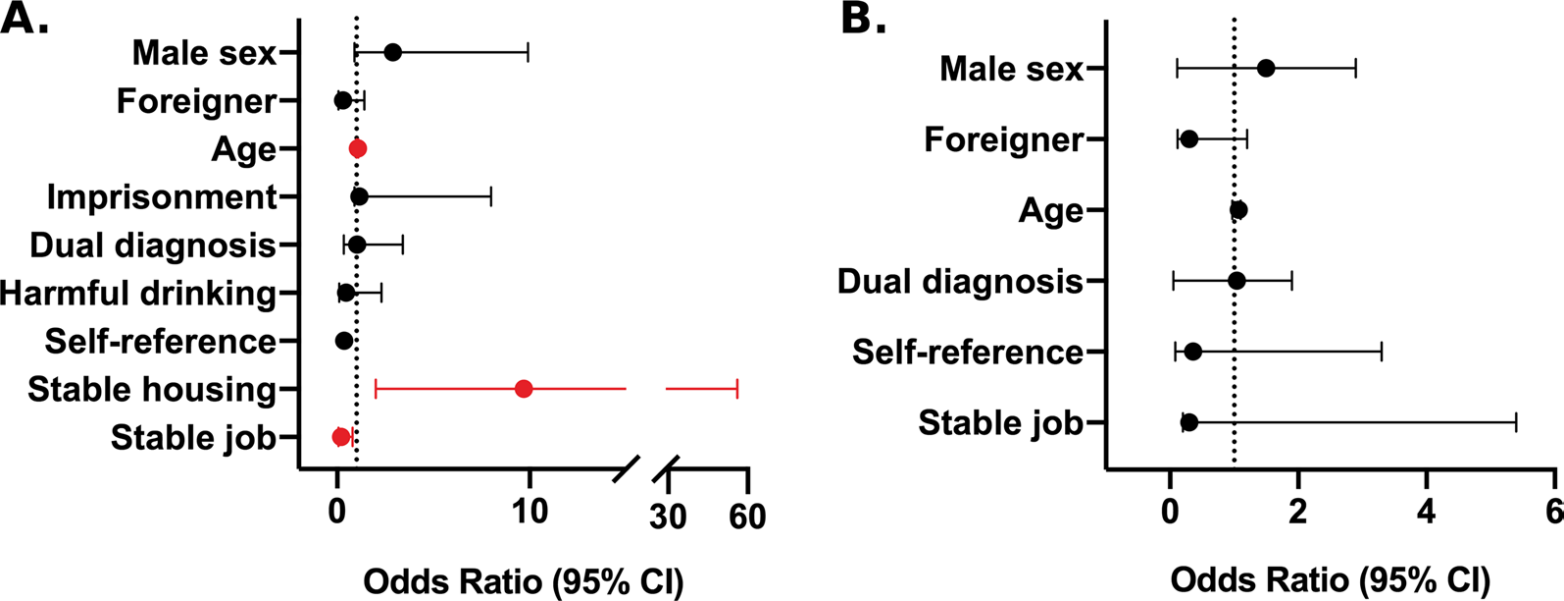
Factors determining adherence to therapy (**A** people who inject drugs group, **B** control group). *CI* confidence interval

Analysis of factors contributing to an absolute adherence to therapy was performed for all patients and for each group separately. In multivariate analysis of the whole cohort of treated patients, IVDU was not a factor contributing to a worse adherence to treatment. Stable housing was a significant predictor of excellent adherence (*p* < 0.05; odds ratio (OR) 5.00, 95% confidence interval (CI) 1.40–19.80), whereas being a foreigner (*p* < 0.05; OR 0.39, 95% CI 0.17–0.94) and self-referred to therapy (*p* < 0.05; OR 0.40, 95% CI 0.17–0.99) negatively influenced adherence to treatment (Fig. 1). In the PWID group, older age (*p* < 0.05; OR 1.07, 95% CI 1.00–1.20) and stable housing (*p* < 0.01; OR 9.70, 95% CI 2.10–56.20) were factors positively contributing to adherence. On the other hand, a stable job was a factor negatively influencing adherence (*p* < 0.05; OR 0.24, 95% CI 0.06–0.81) (Fig. 2A). In the control group, none of the analyzed demographic and social factors had a significant impact on adherence to therapy (Fig. 2B).

## Discussion

In the interferon era, PWID used to be regarded as a special population, difficult to treat, with presumed lower adherence to therapy and lower treatment efficacy [10]. Even though aware of their infection, they were not referred to therapy for different reasons: low treatment adherence, a high risk of treatment-associated adverse events, worse treatment tolerability or a medical contraindication to therapy, i.e., dual diagnosis. These individuals now come to get DAA therapy, but the treatment uptake remains low and treatment accessibility is limited because of many barriers to HCV care in this vulnerable group of patients from the side of physicians, health-care payers and the negative opinion on the reimbursement of therapy by non-professional community [11, 14, 19]. The aim of our study was to support the evidence that the efficacy of therapy in PWID is high and that the financial resources are invested effectively. Achieving cure of people in the epidemiologically high-risk group, we decrease the risk of disease transmission [4, 20, 21].

The above-described group represents a pilot cohort of PWID treated with DAA. Most of the patients reported a long period of abstinence, only a minority of them reported ongoing illicit drug use, and therefore, we assumed a superior adherence to therapy within this subgroup of difficult-to-treat patients to whom antiviral therapy had been denied so far. The aim of this pilot study was to break the myth of PWID non-adherence leading to inferior efficacy of therapy. Our data suggest that treatment is feasible when appropriate methods improving adherence to therapy and motivation are applied, especially in patients with ongoing drug use, and may encourage further centers in successful treatment of PWID and support the willingness to pay for DAA regimens by health insurance companies.

The efficacy of therapy (SVR 12) in the PWID group was 98%, comparable with the control group and with the results of registration trials and real-world studies [22–28]. The results unequivocally justify treatment of PWID. The PWID group patients in our cohort were significantly younger, with a less advanced liver disease. Achieving cure of HCV infection, we can prevent progression of liver disease with future decrease of liver-related morbidity and mortality [4, 29].

Early after the introduction of DAA into routine clinical practice in the Czech Republic, in 2015 and 2016, the rate of patients entering DAA therapy was lower in comparison with recent data. Patients with advanced liver disease, most of them with cirrhosis, organ transplant recipients and patients on maintenance hemodialysis had been prioritized [30, 31]. PWID had still been treated with peginterferon-α or had not been treated owing to medical contraindications, such as dual diagnosis. Over a period of 4 years, the percentage of treatment uptake of PWID has increased and represents nowadays more than 75% of treated patients. There are no more treatment restrictions concerning severity of liver disease, but the only criteria are motivation of the patient and adherence to therapy [32].

We consider the adherence to therapy of our patients to be very good. The adherence to therapy in the PWID group was worse only in the number of missed appointments, not in the use of medication. The patients were aware of the necessity of regular use of medication and came accurately to the appointments when new medication was dispensed. On the other hand, their adherence decreased after SVR 12 visit, after being cured. We attribute this fact to the younger age of PWID patients and the sensation of good health. In the control group, patients were older, more often with cirrhosis, undergoing surveillance of hepatocellular carcinoma; consequently, their motivation in the regular follow-up was higher and medically understandable.

In the PWID, the factors positively influencing adherence were older age and, above all, stable housing. It is in accordance with the fact that “housing first” approach represents one of the most important strategies of social integration of PWID. On the other hand, a stable job was a factor predicting worse adherence, leading to a higher number of postponed and missed visits. In the Czech Republic, HCV infection is still perceived as a highly stigmatizing disease. Most HCV-infected patients are reluctant to confess their diagnosis, being afraid if losing their job. Therefore, this fact is an important obstacle to scheduling and adherence to treatment visits.

Our results, SVR 12 and adherence, are better than in the above-cited studies [15–17]. This fact can be attributed not only to the patients' motivation but also to the support of physicians and nurses. We have had a long-term experience with HCV therapy including PWID group, and we have had a long-term collaboration with low-threshold drug addiction centers, rehabilitation centers and therapeutic communities. Thanks to this collaboration, we pointed out the factors contributing to a better adherence to therapy and every patient had a tailored approach.

In the PWID group, IVDU was presumed to be the most important route of HCV transmission. However, most PWID had been exposed to more than one risk factors for HCV transmission, as amateur tattoo, sexual transmission or imprisonment. They should be aware of other risk factors for HCV transmission which may represent a potential source of reinfection after successful course of therapy. In close collaboration with harm reduction services centers and their employees, in our group of treated patients, the implemented harm reduction strategies included not only providing sterile needles, syringes and other injection equipment, but also education about overdose prevention, safer injection practices, basic health services (including vaccination), referrals for substance use disorder treatment and job and housing counseling services.

## Conclusions

In PWID, treatment efficacy was excellent and was comparable with SVR of the control group. Stable housing and older age were the major factors contributing to a better adherence to therapy.

## Data Availability

The datasets used and analyzed in study are available from the corresponding author by request.

## Abbreviations

CI: Confidence interval
DAA: Direct-acting antivirals
HCV: Hepatitis C virus infection
IVDU: Intravenous drug use
OR: Odds ratio
PWID: People who inject drugs
SVR: Sustained virological response
